# The Impact Analysis of Land Features to JL1-3B Nighttime Light Data at Parcel Level: Illustrated by the Case of Changchun, China

**DOI:** 10.3390/s20185447

**Published:** 2020-09-22

**Authors:** Fengyan Wang, Kai Zhou, Mingchang Wang, Qing Wang

**Affiliations:** 1 College of Geo-Exploration Science and Technology, Jilin University, Changchun 130026, China; wangfy@jlu.edu.cn (F.W.); zhoukai18@mails.jlu.edu.cn (K.Z.); wangmc@jlu.edu.cn (M.W.); 2 College of Construction Engineering, Jilin University, Changchun 130026, China

**Keywords:** JL1-3B, high resolution, nighttime light intensity, land features, parcel level

## Abstract

Nighttime lights (NTL) create a unique footprint left by human activities, which can reflect the economic index and demographic characteristics of a country or region to some extent. It is of great significance to explore the impact of land features related to social–economic indexes to NTL intensity in urban areas. At present, there are few studies on the impact factors of high-resolution NTL remote sensing data to analyze the influence of NTL intensity variation at a fine scale. In this paper, taking Changchun, China as a case study, we selected the new generation of high spatial resolution (0.92 m) and multispectral bands NTL image JL1-3B data to evaluate the relationship between NTL intensity and related land features such as the normalized difference vegetation index (NDVI), land use types and point of information (POI) at the parcel level, and combined Luojia 1-01 images for comparative analysis. After screening features by the Gini index, 17 variables were selected to establish the best random forest (RF) regression model for the Luojia 1-01 and JL1-3B data, corresponding to out-of-bag (oob) scores of 0.8304 and 0.9054, respectively. The impact of features on NTL was determined by calculating the features contribution. It was found that JL1-3B data perform better on a finer scale and provide more information. In addition, JL1-3B data are less affected by light overflow effect and saturation, and they could provide more accurate information at smaller parcels. Through the impact analysis of land features on the two kinds of NTL data, it is proven that JL1-3B images can be used to study effectively the relationship between NTL and human activities information. This paper aims to establish a regression model between the radiance of two types of NTL data and land features by RF algorithm, to further excavate the main land features that impact radiance according to the feature contribution, and compare the performance of two types of NTL data in regression. The study is expected to provide a reference to the further application of NTL data such as land feature inversion, artificial surface monitoring and evaluation, geographic information point estimation, information mining, etc., and a more comprehensive cognition of land feature impact to urban social–economic indexes from a unique perspective, which can be used to assist urban planning and related decision-making.

## 1. Introduction

With the continuous development of the scale and scope of human activities, night lighting facilities have gradually become popular, and human beings have gradually removed the darkness from night [1]. The nighttime lights (NTL) imagery obtained by remote sensing technology provides a unique perspective and way for the observation and analysis of human night activities from space [2,3,4]. Hence, the research process of human activities and urbanization can be effectively supported by large-scale and long-term NTL observation. 

In general, the NTL intensity is strongly correlated with socio-economic indicators [5,6] such as population [7,8,9], gross domestic product (GDP) [10,11,12,13,14,15], electricity consumption estimates [16,17], house vacancy [18,19], dynamic monitoring [20,21], carbon emission estimation [22,23,24], fishery studies [25,26,27,28], and poverty and Gini coefficient estimates [29]. Furthermore, due to cities shining intense light at night, NTL imagery plays an important role in monitoring the process of urbanization [30,31,32] and the extraction of built-up areas [33,34,35,36,37,38,39,40]. Table 1 shows the parameters associated with common NTL data.

At present, when using NTL data for analysis and research, most of them are still based on the macroscopic scale such as the extraction of built-up areas, local economic estimation, and population correlation, and NTL data are more used as auxiliary data for research. In order to further explore the relationship between NTL data and socio-economic indicators, it is of great significance to find the corresponding land features that cause the change of NTL and the extent of impact by the method of fusing multi-source data. It is of great research significance to find the land features that have important impact on the radiance of the high-resolution NTL image and calculate the impact of these land features. Li et al. used two kinds of NTL data obtained by Operation Linescan System on Defense Meteorological Satellite Program (DMSP/OLS) and National Polarorbiting Partnership on Visible Infrared Imaging Radiometer Suite (VIIRS/NPP), respectively to analyze the impact of land use types to the NTL radiance [41]. Given the low resolution of NTL images, different land use types are mixed in each pixel, so an unmixing model was proposed to quantify the contribution of land features to coarse-resolution NTL images. This was easily restricted by the data resolution, and it only analyzed the impact of land use types to NTL intensity. Ma studied the relationship between VIIRS-DNB night light data and land use types and POI (point of information) density at the pixel level [42]. The results showed that most urban and built-up pixels are lighted and many non-man-made surfaces are also lit in current NTL images mainly because of the light overflow effect, which means the detected light range reflecting human activities is often larger than the actual situation. Although the NTL intensity showed a positive linear correlation with the corresponding POI density, only a small percentage of it could be explained by the spatial variation of VIIRS-DNB NTL radiances. In addition, with limited spatial resolution, some small built-up areas far from urban centers may not be captured by images. Therefore, the research scale gradually improved on a single pixel to a parcel level. In modeling residential developed land in rural areas, Leyk et al. chose the parcels as the research scale, and the study showed that the information on the parcel level could better reflect the land performance compared to the pixel level [43]. Chen et al. analyzed the relationship between urban expansion and DMSP/OLS NTL data at the parcel level [44]. In addition, Chen also introduced POI data in research to find that different types of POI have different effects on the strengths and weaknesses of NTL data—for example, shopping malls—and that enterprises’ POI points are more affected than in residential areas and factories. The results show that the calibrated NTL data create a reliable index that reflects the combined effect of integrated human activities. Wang et al. applied POI data and land use data to establish regression models with Luojia 1-01 and VIIRS/NPP, and analyzed the influence degree of different features [45]. The study showed that Luojia 1-01 has a higher dynamic range across different land use types than VIIRS and had a higher potential for distinguishing urban area from other land use types.

A large number of studies show that the intensity of NTL is closely related to land features and human activities. The study of the impact of land features on the radiance of NTL images can provide a reference for future applications such as land feature inversion, artificial surface monitoring and evaluation, geographic information point estimation, and information mining using high-resolution, multispectral NTL images. However, the resolution of NTL data used in most studies is over 100 m, making it difficult to meet the requirements of fine information extraction in the region. As the first NTL data that can be provided with multiple spectra (blue, green and red), JL1-3B data have a resolution of 0.92 m, which is a very high resolution, and extremely rich spectral band information; as a result, the study can be carried out on a more refined scale, such as the extraction of street light information [46], and the data are also used to further improve the accuracy of the study sample.

To sum up, in order to further evaluate and apply JL1-3B NTL data with the high-resolution and multispectral capability, it is necessary to clarify the relationship between the radiance of NTL data and land features. We established a random forest (RF) regression model between the radiance of NTL data and land features at the parcel level and analyzed the feature contribution of different land features. Through the comparison with previous relevant studies, there are three improvements in this paper: data quality, land feature selection, and the log processing of the regression values. The contribution of the study is reflected in that the RF regression model between the radiance of NTL data and land features at the parcel level was proposed and established with a good fitting effect, which can provide a reference model and method for the evaluation of NTL data; the analysis of the feature contribution variation of different land features is conducive to grasp the key impact factors of NTL intensity and provide a reference for the NTL data application; it is verified that JL1-3B data can be effectively used to study the relationship between NTL and human activities, and it has broad application prospects in the future.

## 2. Study Area and Data Sources

### 2.1. Study Area

**Figure 1:**
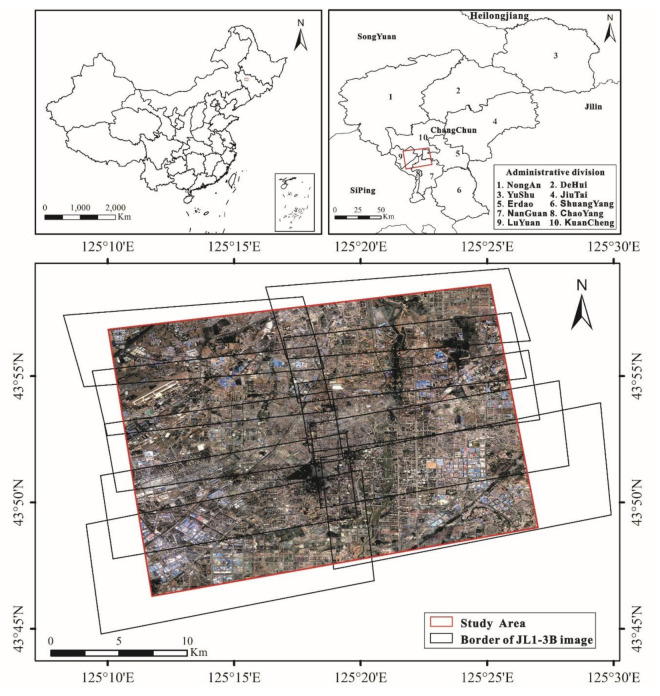
The basic information of the research area, superimposed on the Google Earth image. The red and black regions represent the border of the study area and each part of the JL1-3B data, respectively.

Changchun (43°05′ N to 45°15′ N, 124°18′ E to 127°05′ E) is the capital city and the political, economic, cultural, and transportation center of Jilin province in Northeast China, which is also an important industrial and commodity grain base in China. It is the center of the Northeast-Asia Economic Cycle (Figure 1). The humid continental climate with long, cold winters governs the regional biomes, which are represented by temperate conifer forests. After the reform and opening up of the country, Changchun gradually formed a service industry and commercial spaces, and it developed rapidly since the early 1980s [47]. In 2014, Changchun’s gross domestic product (GDP) reached 861.12 billion RMB Yuan, and its per capita disposable income reached 11335.85 RMB Yuan. As of 2018, the jurisdiction of Changchun has seven districts and three countries, with a total area of 20,593.5 km^2^ and a built-up area of city is 660.19 km^2^. Over the past few decades, the built-up area of Changchun has been increasing continuously, and the city has been developing in an orderly direction. To achieve various goals of sustainable urban development and structural transformation, it is of great significance to understand the impact of some typical land features on urban social and economic parameters. Therefore, we take the NTL intensity as the indicator reflecting the economy and central Changchun as the study area. As shown in Figure 1, the study area covers each part of five districts of Changchun: Eradao, Nanguan, Kuancheng, Chaoyang, and Luyuan.

### 2.2. Datasets

Table 2 shows details of the data used in this study, including two kinds of NTL data: JL1-3B and Luojia1-01, land use data (GLOBALAND30), POI, road networks data (Open street map), and normalized difference vegetation index (NDVI) products generated by using Landsat8 Operational Land Imager (OLI) images.

#### 2.2.1. NTL Data

The JL1-3B satellite, a solar-synchronous orbiting satellite with an orbital altitude of 535 km, was developed and launched by Chang Guang satellite technology Ltd. and began commercial operation on 1 August 2017. The minimum detectable radiation value of the JL1-3B satellite is $7\times 10^{-7}$ W/cm^2^/sr. Compared with other common NTL data, JL1-3B data have three advantages: (1) higher resolution (0.92 m); (2) three different bands including a blue band (437–512 nm), green band (489–585 nm), and red band (580–723 nm); and (3) on-board radiation calibration [48]. Such a high spatial resolution allows JL1-3B data to be used for more fine-scale land use studies [49], as well as for the classification of light sources.

The Luojia satellite is a scientific experimental engineering satellite launched on 2 June 2018, and its main mission is to obtain NTL data and to conduct experimental validation of low-orbit satellite navigation enhancements. Luojia-1 is a solar-synchronous orbiting satellite that can cover the globe in 15 days. The Luojia 1-01 data are NTL imagery obtained by the high-resolution night light sensor on the Luojia-1 satellite, with a ground resolution of 130 m and a width of 250 km per image. By May 2019, the satellite imagery had fully covered China and some parts of Southeast Asia [50]. Luojia 1-01 data have a high resolution and rich spatial information, and compared with previous NTL images such as DMSP/OLS, VIIRS/DNB, and other images, the data can obtain better identification results than the predecessors [51].

**Figure 2:**
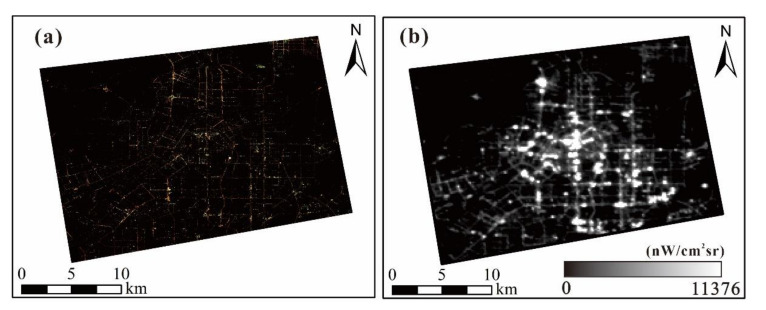
Two kinds of nighttime lights (NTL) data in the study area: (**a**) JL1-3B; (**b**) Luojia 1-01.

In this study, the Luogia 1-01 data and JL1-3B data were regarded as regression objects, of which one single image of Luojia 1-01 was taken on August 16, 2018, and ten images of JL1-3B were obtained on April 25, 2018. Figure 2 presented JL1-3B data and Luojia 1-01 data, which have the same range as the Google image shown in Figure 1.

#### 2.2.2. Road Networks Data

**Figure 3:**
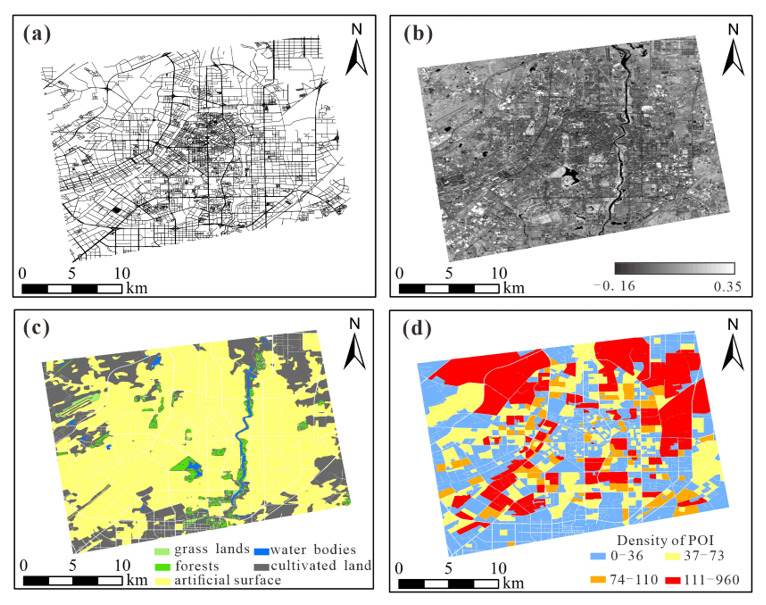
Distribution of land features data in parcel level: (**a**) road networks (Open Street Map, OSM); (**b**) normalized difference vegetation index (NDVI); (**c**) land use data; (**d**) density of point of information (POI).

The road networks data of Changchun were collected from Open Street Map (OSM). OSM provides open and free geographic data with different levels of road vector data networks. The combination of road networks of appropriate levels can be regarded as a natural division boundary [52]. After screening, there were six types of road networks used in this study: highways, main roads, primary roads, secondary roads, tertiary roads, and residential roads; the corresponding road widths are 30 m, 30 m, 20 m, 15 m, 10 m, and 8 m respectively, as illustrated in Figure 3a.

#### 2.2.3. Data Reflecting the Land Features

Different land use types will correspond to different levels of NTL intensity. We used global land use data provided by the National Center for Basic Geographic Information (GLC30), which is based on the 30 m spatial resolution. GLC30 was obtained by visual interpretation and field verification with high precision-based on Landsat, ETM+, and China Environmental Disaster Reduction Satellite (HJ-1). The data include 10 types of land use such as cultivated land, forests, grasslands, shrubland, wetlands, water bodies, tundra, artificial surfaces, bare land, glaciers, and permanent snow. After clipping, in the study area, there are five kinds of land use types: cultivated land, forests, grass lands, water bodies, and artificial buildings. Figure 3c shows the distribution of five kinds of land use.

As geographic information reflecting human activities, POI also has a certain influence on night light intensity. The POI data were obtained from Baidu Map (http://lbsyun.baidu.com). The study area contains 59,897 POI data of 16 types: street furniture, place and address information, tourist attraction, enterprises, shopping services, road ancillary facilities, financial insurance services, science and education, food, automobile services, automobile maintenance, automobile sales, business housing, life services, medical care services, and government agencies. After data cleaning, the coordinates are transformed and projected to the Universal Transverse Mercartor (UTM) projection. Then, we generated a POI density map in which every unit pixel value means the numbers of POIs in that pixel. Figure 3d shows the parcel level density of POIs.

In addition, Levin et al. found that the NTL intensity of a city will change with the seasons [53], which means that the *NDVI* of different parcels has a certain influence on the corresponding radiance. Therefore, we introduced *NDVI* as an indicator of vegetation conditions. The *NDVI* can be calculated as follows:

(1) $$NDVI=\frac{NIR-R}{NIR+R}$$

where *NIR* and *R* are the reflectance of the infrared band and red band. The cloud coverage of all the Landsat8 images (the paths and rows of images are 30 and 118, respectively) taken in the same month (April 2018) as JL1-3B is 100%. Therefore, cloud-free images taken at the same position in the same month (April 2) in 2017 were selected to obtain NDVI data through band combination calculation after radiance correction and atmospheric extinction (as illustrated in Figure 3b).

## 3. Methods

**Figure 4:**
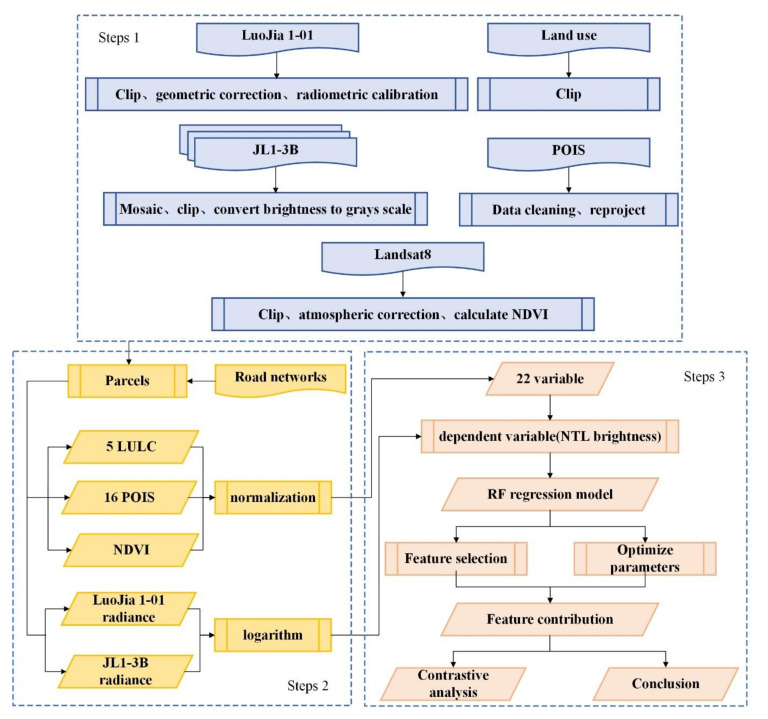
Methodology flowchart. Step 1: data preprocessing; step 2: making parcels datasets; step 3: establishing the random forest (RF) regression model and analyzing the features contribution.

By constructing a regression model between the radiance of JL1-3B data and related influencing factors, and using Luojia 1-01 data for comparison, this paper studied the relationship between the NTL intensity and land features, as well as the performance of the two kinds of data in different aspects during the regression process. The research process as shown in Figure 4 includes three major steps: (1) data preprocessing; (2) making experimental datasets; (3) establishing the regression model, and making a comparative analysis of the results.

### 3.1. Data Preprocessing and Experimental Datasets Making

**Figure 5:**
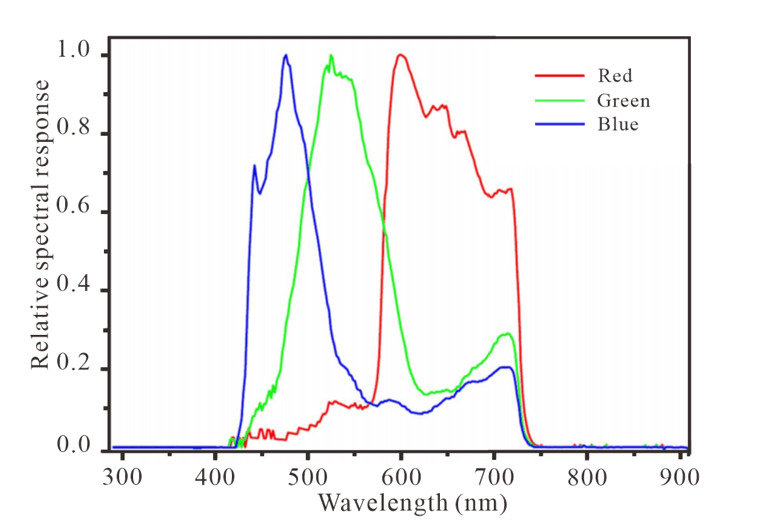
The RGB relative spectral response of JL1-3B.

All the datasets were clipped with the boundary of the study area and reprojected to the Mercator (UTM) projection of the WGS-84 coordinate system. Due to the Luojia 1-01 NTL images having slight geo-referencing errors, a geometric correction was performed based on Google Earth imagery. To ensure that the two kinds of NTL data can be compared, we need to convert the DN value to radiance for two kinds of NTL data. According to Equation (2), the DN value of the Luojia 1-01 image can be converted to the radiance.

(2) $$R_{1}=10^{-10}D^{3/2}$$

where *R*_1_ represents the radiance (W/m^−2/^sr^−1^) of each pixel, and *D* represents the Digital Number (DN) value of image. Different from normal NTL data, JL1-3B is a multispectral image and cannot directly quantify radiance. Figure 5 shows the spectral response curve of JL1-3B data. First, the DN value of each band was converted to radiance according to Equation (3):

(3) $$DN=aR_{2}+b$$

where *R*_2_ is the radiance, and *a* and *b* are corresponding parameters, as shown in Table 3.

In this paper, the radiance of three bands of JL1-3B data was combined to $R_{2}^{*}$ according to Equation (4) [54].

(4) $$R_{2}^{*}=0.2989\times R_{2\_red}+0.5870\times R_{2\_green}+0.1140\times R_{2\_blue}$$

where *Red*, *Green*, and *Blue* are the different radiances of each band. Then, buffers were established for different levels of road networks data according to road width. After removing road spaces, 3057 land parcels were generated, and the feature information from each parcel was extracted. For the convenience of established random forest regression, all the features were normalized by using Equation (5):

(5) $$X_{nor}=\frac{X_{i,j}-\min(X_{i})}{\max(X_{i})-\min(X_{i})}$$

where *X_nor_* is the normalized value for feature *i* at parcel *j*; *X_ij_* is the value at parcel *j*; *X_i_* is variable *i;* and *X_j_* is variable *j.* In addition, the logarithms of two kinds of NTL intensity were taken to facilitate computations.

### 3.2. Random Forest (RF) Regression Model

The RF model is a kind of fusion algorithm based on a decision tree. It uses a large number of weak predictors to integrate into a strong predictor to improve the regression result [55]. The RF model is mainly applied to the fields of classification and regression. As a nonlinear modeling algorithm, the RF regression model is widely used in data mining [56], bioinformatics statistics [57,58], and other fields. Many studies have proved that RF regression has very high prediction accuracy, and it is less affected by noise and outliers, so it is not easy to overfit [59]. In this paper, the regression method of random forest was selected to analyze the impact of different land features on the radiance in detail, and the feature contribution was taken as the index to reflect the impact of different features. The out-of-bag (oob) score was used as the evaluation index to evaluate the outcome of the regression.

**Figure 6:**
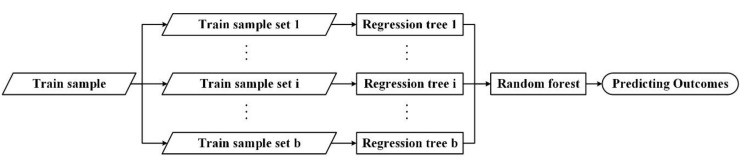
Random forest regression procedure.

Similar to most models, RF regression is also intended to establish relationships between several independent and dependent variables. Assume that the dependent variable *Y* is affected by *k* independent variables. In the process of constructing the regression tree, the RF will use a bootstrap resampling method to randomly sample some observations of the dependent variable *Y*, and randomly select a specified number of variables from the *k* independent variables to determine the regression tree node. This ensures that the regression tree constructed each time is different due to randomness. Then, among the hundreds of regression trees generated, the tree with the highest degree of repetition is selected as the final result. The combined model formed by the regression trees can be described as $\left\{h\left(X,\theta_{j}\right),\;j=1,2,\cdots ,b\right\}$, where θ is the regression tree, and *j* is the serial number of the regression trees. The predicted value of the RF regression model is formed by calculating the average value of all the regression trees. The *RF* regression procedure described above is shown in Figure 6. The satisfying condition of the model is that the multiple training datasets forming the RF regression are independent of each other.

The flow of the RF regression algorithm is as follows:

(1) From the *n* observations in the original datasets, we apply the bootstrap method to repeatedly extract *b* training sample sets and construct *b* regression trees. In addition, *b* samples of out-of-bag (oob) data composed of unextracted observations are used as the test datasets.
(2) When constructing the regression trees, randomly select *m* (*m* < *k*) candidate branch variables from *k* independent variables at the branch node of each tree, and then determine the optimal branch in it according to the optimal branch criterion.
(3) Each tree branches recursively from top to bottom and grows continuously.
(4) Generating b regression trees constitutes a RF regression model. One of the advantages of RF is that there is no need to cross-validate it or use an independent test set to obtain an unbiased estimate of the error. It can establish an unbiased estimate of the error during the generation process of trees. When each decision tree is generated, samples are drawn randomly and replaced. For each decision tree, about 36.8% of the samples are not drawn. These samples are called the out-of-bag data of each tree. This part of the data is not involved in the construction of the decision tree, so it can be used to evaluate the outcome of the regression model. The oob data of each tree is not the same. In this paper, the $R^{2}$ between the predicted value and the true value of all oob data is the oob score of the regression model. The effect of model estimation is measured by the mean square error of oob data prediction according to Equations (6) and (7):

(6) $$MSE_{OOB}=\frac{\sum_{i=1}^{m}{\left(y_{i}-{\hat{y}}_{i}\right)}^{2}}{m}$$

(7) $$R^{2}=1-\frac{MSE_{OOB}}{{\hat{\sigma }}_{y}^{2}}$$

where $y_{i}$ is the true value of the dependent variable, ${\hat{y}}_{i}$ is the predicted value obtained by the RF regression model, and ${\hat{\sigma }}_{y}^{2}$ is the variance of the oob predicted value.

Normally, only a few features that have a greater impact on the results will participate in the final modeling process. A major feature of the ensemble learning model is that it can output feature importance, which can assist us in screening features to a certain extent, so that the model is more robust. The idea of evaluating the features importance in RF is to judge how much each feature contributes to each tree and then compare the features’ importance based on the mean of each of them. One of the methods to assess the importance of features in RF is based on the Gini index [55] according to Equation (8):

(8) $$Gini\left(p\right)=\;\overset{K}{\underset{k=1}{\sum }}p_{k}\left(1-p_{k}\right)$$

where *k* is the number of variable types, and $p_{k}$ is the sample weight for category *k*. If the node of feature $X_{j}$ in regression tree *i* is the set *M*, then we can calculate variable importance measures (VIM) of $X_{j}$ in the *i*-th tree according to Equation (9):

(9) $$VIM_{ij}^{\left(Gini\right)}=\sum_{m\in M}VIM_{jm}^{Gini}.$$

In terms of a random forest with *n* regression trees, the VIM of $X_{j}$ is according to Equation (10):

(10) $$VIM_{j}^{\left(Gini\right)}=\sum_{i=1}^{n}VIM_{ij}^{\left(Gini\right)}.$$

Finally, we normalized all the obtained importance scores according to Equation (11):

(11) $$VIM_{j}=\frac{VIM_{j}}{\sum_{i=1}^{c}VIM_{i}}$$

where the denominator is the sum of the information gain of all features, and the numerator is the Gini index of feature *j*.

In addition, in order to provide an explicit interpretation to the established RF regression model, the contribution of features could be calculated. As far as decision trees are concerned, each decision made by a tree (or forest) can be regarded as one or more paths from the root to the leaves of the tree, consisting of a series of decisions, and each feature in the path has a contribution to the final forecast. Since each decision is guarded by features, and the decision either adds or subtracts from the value given in the parent node, the prediction can be defined as the sum of the feature contributions and the “bias” (i.e., the mean given by the topmost region that covers the entire training set), as shown in Equation (12):

(12) $$f\left(x\right)=c_{full}+\overset{K}{\underset{k=1}{\sum }}contrib\left(x,k\right)$$

where $f\left(x\right)$ is the prediction, $c_{full}$ is the value at the root of the node, *K* is the number of features, and $\mathrm{contrib}\left(x,k\right)$ is the contribution from the *k*-th feature in the feature vector *x*. For the decision tree, the contribution of each feature is not a single predetermined value, but it depends on the rest of the feature vector, which determines the decision path that traverses the tree and thus the guards/contributions that are passed along the way.

We used scikit-learn, a machine learning tool based on Python, for modeling and analysis. The land use types, the POI density of different types, and NDVI were taken as feature quantities, while the radiance from Luojia 1-01 and JL1-3B is taken as the regression quantity, and the oob score is taken as the evaluation index. After feature screening and adjusting parameters, an optimal RF regression model is established, and its influence on radiance was analyzed according to the features contribution.

## 4. Results

### 4.1. Radiance Variations with Land Features

**Figure 7:**
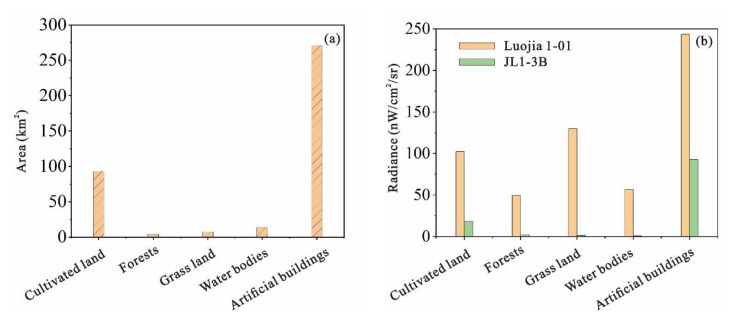
(**a**): The area corresponding to different land use types. (**b**): The average radiance of the Luojia 1-01 and JL1-3B data corresponding to different land use types.

Before the regression, by calculating the area of each land use and the corresponding average NTL intensity, we can initially understand the relationship between different land use types and NTL intensity, as shown in Figure 7. Since the study area is mainly located in the central area of the city, the area of artificial buildings and cultivated land is 271.2 km^2^ and 93.2 km^2^, respectively, accounting for a relatively high proportion, 69.45% and 23.89%, respectively. It can be seen from Figure 7 that the average radiance corresponding to artificial buildings is the highest. In contrast, JL1-3B data have higher resolution, less light saturation, and better separation of city lights, etc. Therefore, the radiance corresponds to different features, which are more consistent with the actual situation. The average radiance of the Luojia 1-01 data corresponding to different ground cover types is higher than that of the corresponding JL1-3B data due to the light overflow effect and the oversaturation of city lights, etc. Therefore, a JL1-3B image more realistically represents lighting conditions on the ground and has a better separation of light sources. For example, if the light emitted by a certain light source, such as street lamps on a building or a road, is very strong, which is reflected in Luojia 1-01 images that are beyond a certain range of strong light sources, dark regions actually appear as existing radiance, which is not obvious in JL1-3B images. This results in the radiance corresponding to Luojia 1-01 data being higher than that corresponding to JL1-3B data under the same land use type. This also results in areas where radiance should be low, such as cultivated land, grassland, and water bodies, but where the average radiance of Luojia 1-01 remains high.

### 4.2. RF Regression Fitting

When establishing the RF regression relationship between the parcel level correlation features and the NTL intensity at the parcel level, in order to avoid introducing uncorrelated or low-correlation features, different features are screened to remove some features with minimal effect. In this paper, with Gini index as the feature importance, all 22 features are ranked, and the features with the lowest importance are removed one by one. At the same time, the out-of-bag score was calculated until the out-of-bag score did not increase, or even decreased, and feature screening was finished. The features initially selected in this study include five land use types (cultivated land, forest, grassland, water, and artificial buildings), 16 POIs (street furniture, place and address information, tourist attraction, enterprises, shopping services, road ancillary facilities, financial insurance services, science and education, food, automobile services, automobile maintenance, automobile sales, business housing, life services, medical care services, and government agencies), and NDVI. In the process of feature selection, after removing the five POI features with the lower importance such as street furniture, location and address information, tourist attractions, business housing, and medical and health services in turn, the out-of-bag score reached the highest, so the 17 features were finally selected to participate in the regression process. The corresponding feature importance is shown in Table 4.

It can be seen that the feature importance of artificial surfaces is the highest in the regression of the two kinds of NTL data of JL1-3B and Luojia 1-01, which are 70.04% and 69.42%, respectively. In GLOBALAND30 land use data, the artificial surface specifically refers to the surface formed by artificial construction activities, including various types of residential areas such as cities and towns, industrial and mining, transportation facilities, etc., but it does not include the continuous green space and water bodies within the construction land.

For the POI types in the regression of JL1-3B data, the POI category with the highest feature importance is the food, which is 0.8%, while the regression of the Luojia 1-01 data has the highest feature importance, 3.16%. The NDVI in the JL1-3B data and the regression of Luojia1-01 are third in the order of feature importance, which are 3.09% and 8.12%, respectively. In general, the importance of different land features in the regression models of the two kinds of NTL data is somewhat consistent.

After parameters testing, the optimal parameters of the regression model of JL1-3B data and Luojia 1-01 are shown in Table 5, and the final oob scores are 0.9054 and 0.8304, respectively.

### 4.3. Contribution Analysis of Significant Features for RF Regression Model

**Figure 8:**
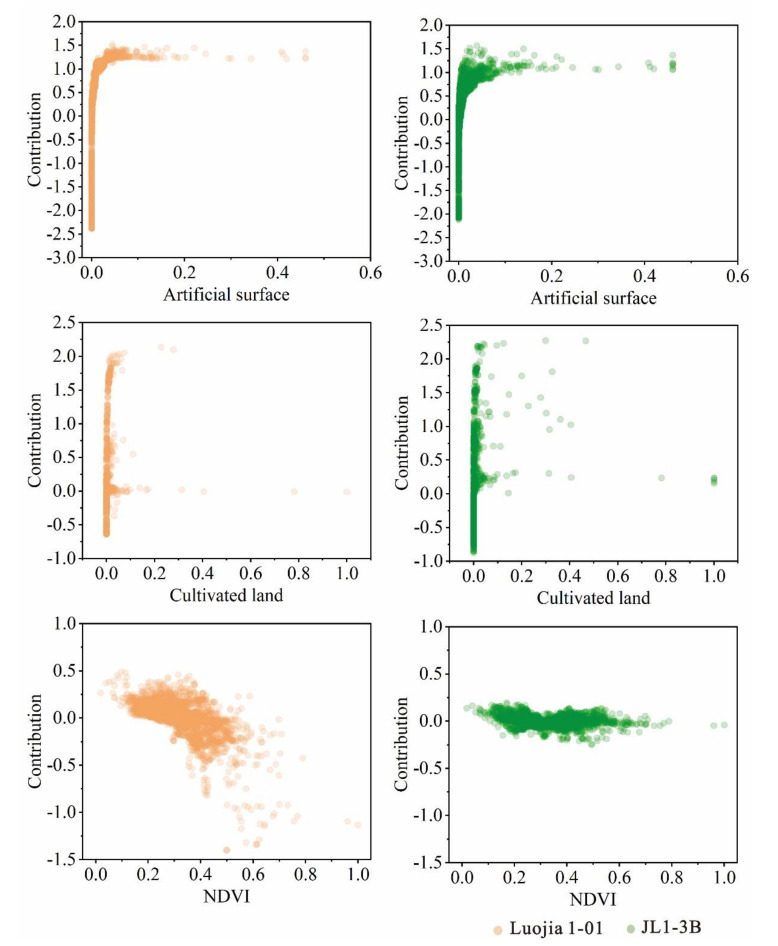
The features contributions of artificial surface, cultivated land, and NDVI in the RF models for Luojia1-01 and JL1-3B data.

**Figure 9:**
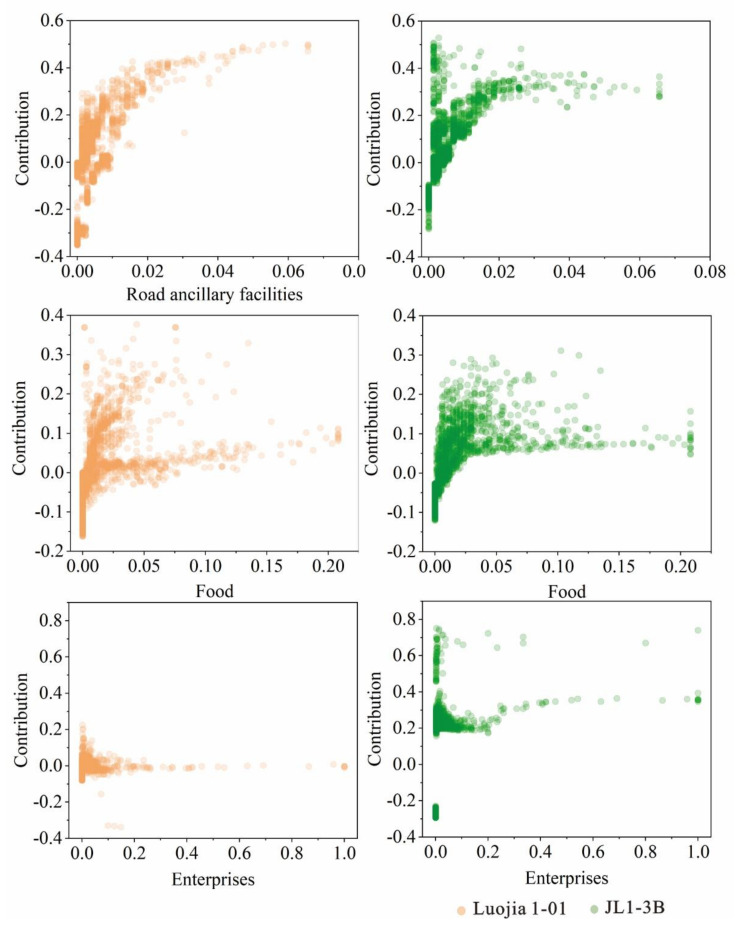
The features contributions of road ancillary facilities, enterprises, and food in the RF models for Luojia1-01 and JL1-3B.

Figure 8 and Figure 9 show the corresponding relationship between the contribution values of some significant features variables and themselves, which revealed the contribution of different features to different degrees in the RF regression model. The larger the contribution, the greater the impact on NTL radiance. According to the principle of features importance, several relatively important features are selected for analysis under different features, including two land use types (cultivated land and artificial surfaces), three POI types (road ancillary facilities, enterprises, and food), and NDVI. In order to ensure that the results of regression models of two kinds of NTL data were comparable, all the data of different features were normalized. For land use types, the normalized object is the area corresponding to different land use types in each parcel; for POI, the normalized object is the number of points corresponding to different POI types in each parcel; for NDVI, the normalized object is the average value of the NDVI in each parcel.

It can be seen that the contribution of artificial buildings from land use type to NTL intensity is basically positive, specifically between −2.0 and 1.6. With the increase of artificial surface area, the contribution value first increased rapidly and then stabilized at about 1.2. The variation range of the contribution value of the cultivated land area is larger when the area is small, specifically between −0.7 and 2.4, but it basically tends to 0 with the increase of the area. This is because different parcels are divided by road networks, so the parcels with small areas are mostly located in the concentrated sections of roads. Even though the land features are completely cultivated, the parcels with small areas are still illuminated with certain radiance due to the surrounding road lights, but once the area is enlarged, the parcels will appear in a dark state. In terms of NDVI, the contribution value ranges from −1.5 to 0.5. It can be seen that compared with JL1-3B data, the contribution of Luojia 1-01 has a larger range of change, and the contribution gradually becomes negative in the process of enlargement as the NDVI changes from negative to positive. This is because the higher the NDVI value, the higher the probability of vegetation cover, and the lower the probability of NTL existing in the same spaces, so the contribution to the radiance regression of the site is negative, which indicates that Luojia 1-01 data are more sensitive to NDVI changes in the regression compared with JL1-3B data.

Changes in the contribution values of the three representative features variables related to POI are as shown in Figure 9. It can be seen that the variation trend of the contribution value of the three POI data of road ancillary facilities, enterprises, and food in the two NTL data regression models is basically the same, and the variation ranges are −0.4 to 0.6, −0.4 to 0.8, and −0.2 to 0.4, respectively. However, the JL1-3B data can provide more nuanced information about the changes. In particular, in the case of a small number of POI, the regression for JL1-3B data still has a high contribution value compared to the Luojia 1-01 data.

It can be seen that the change range and trend of the two land features of land use types and POIs for the two kinds of data are basically consistent. However, the feature contributions show a steady state only when the value of the X-axis is above a certain value. When the value of the X-axis approaches 0, it does present obvious uncertainty, which is manifested in a large variation range of corresponding contribution. This means that in the process of regression, if the amount of information of a feature is low (for example, the area of a certain land use feature or the point number of a certain POI accounts for a small proportion), the contribution of the feature will be difficult to determine, and the contribution of those scattered points under the steady trend usually represents the general situation of this feature at the parcel level. Therefore, the contribution of three types of land features was analyzed after ignoring the uncertainty point. For land use features, the contribution of artificial surface to JL1-3B and L1-01 in land features is about 1.2 and 1.1, respectively, and the contribution of cultivated land to L1-3B and L1-01 is about 0.1 and 0.3, respectively. For POIs, the contribution of road facilities to JL1-3B and Luojia 1-01 is about 0.4 and 0.3, respectively; the contribution of good food to JL1-3B and Luojia 1-01 is about 0.06 and 0.03, respectively; and the contribution of enterprises to JL1-3B and Luojia 1-01 is about 0 and 0.3, respectively. In addition, as can be seen from Figure 8, for NDVI, the scatter points with negative contribution are more than those with positive contribution. The higher the value of NDVI, the higher the proportion of vegetation coverage, and the lower the probability of the existence of lights will be, which is reflected in the negative contribution in the regression. Moreover, Luojia 1-01 data are more sensitive to vegetation change compared with JL1-3B data.

## 5. Discussion

### 5.1. Regression Results Analysis of Two NTL Data

Taking Changchun, China as a study area, choosing land use types, POI density, and NDVI as a land feature, a RF regression model was established to reveal the impact of these land features to JL1-3B NTL data at the parcel level and compare the results with Luojia 1-01 data. There are three improvements in this paper: data quality, land feature selection, and the log processing of the regression values through the comparison with previous relevant studies.

**Figure 10:**
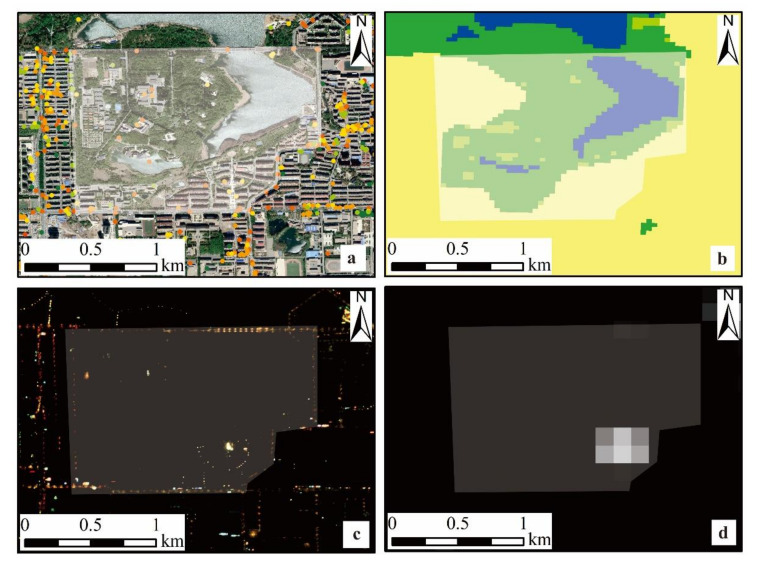
A sample of one of the parcels located in Nanhu Park superimposed on different types of features and NTL data: (**a**) POI superimposed on Google map image taken on May 6, 2018. Different colors represent the number of points of different POI types. (**b**) Land use. (**c**) JL1-3B. (**d**) Luojia 1-01. The shaded region represents the border of the parcel generated by OSM.

In terms of data quality, we selected the light radiance of JL1-3B data and Luojia 1-01 data as regression objects. Figure 10 is a representative sample of one of the parcels in the study area. Generally, it is hard to verify the actual radiance sources of multifunctional buildings, which may comprise different land use types and take on different construction applications, especially in urban areas. So, it is necessary to choose a preferable scale to ensure that it contains a lot of relevant features information. That is why parcels are more suitable than pixels to analyze the relationship between human activity features and NTL radiance on fine spatial scales [60].

By comparing the regression models of different NTL data, there are obvious differences in the average radiance corresponding to different kinds of land features, and this difference is more significant in JL1-3B data than in Luojia 1-01 data. In addition, owing to the great advantage of resolution, compared with the Luojia 1-01, the light overflow effect in JL1-3B is not obvious, which made the former more accurate in finding the radiance corresponding to its land features. It also ensured a high separation of radiance between different light sources, especially at the junction of artificial construction areas and other land use types. In the regression models of the two kinds of NTL data, the contribution values of different variables are basically the same, among which artificial buildings have the strongest impact on the radiance, followed by cultivated land. However, as can be seen as Figure 10, the feature contribution variation of JL1-3B data is more realistic.This also indicates that the JL1-3B data have great potential in the extraction of built-up areas and other fields.

In addition, JL1-3B data provide a more detailed measurement of demographic and social factors. Different POI types have different importance for radiance. Both Luojia 1-01 and JL1-3B can detect rich details of human activity characteristics. In contrast, the contribution of POI data to the radiance of Luojia 1-01 is slightly greater than that of JL1-3B data. In the POI category, enterprises, delicacies, and road ancillary facilities are of great importance to the NTL data, which is conducive to an in-depth analysis of the relationship between human activity elements and the social economy. By comparing the regression of these two kinds of NTL data, the advantages and disadvantages of these two kinds of data are analyzed. Luojia 1-01 has wider data coverage and more convenient access methods. It has a certain potential when conducting research in a relatively large range. JL1-3B has extremely high data resolution, and as a multi-spectral sensor, compared with only one panchromatic band of DMSP-OLS, VIIRS and Luojia 1-01 data can provide richer spectral information.

Different from previous studies in feature selection, NDVI was introduced as one of the land features except for land use types and the density of different types of POI. As can be seen from the result, among feature importance, NDVI ranks third, behind artificial surfaces and cultivated land. This means that NDVI has a relatively large impact on NTL intensity. According to the spectral features of vegetation, NDVI can reflect the growth status of land surface vegetation. The larger the NDVI, the better the plant growth. There are many trees in the park, as shown in Figure 10a, so the mean NDVI of this parcel is relatively high. As we can see from Figure 10c,d, in the forest part of this parcel, there is no light in the Luojia 1-01 image, while there is still abundant light information in the JL1-3B image. This is also the reason why there is a significant difference in the contribution of NDVI to the two NTL data shown in Figure 8.

In processing of the regression values, we performed logarithmic processing on NTL intensity. As the independent variable increases, the variance of the dependent variable also increases. Logarithmic transformation can make the fluctuation of data relatively stable and narrow the threshold range of the dependent variable to facilitate modeling. Without logarithmic processing, the oob scores of the RF regression models JL1-3B and Luojia 1-01 are about 80% and 75%, but the oob scores of the RF regression models JL1-3B and Luojia 1-01 are about 90% and 83% under logarithmic treatment, which increased by 10% and 8%, respectively.

### 5.2. Limitations and Future Prospects

**Figure 11:**
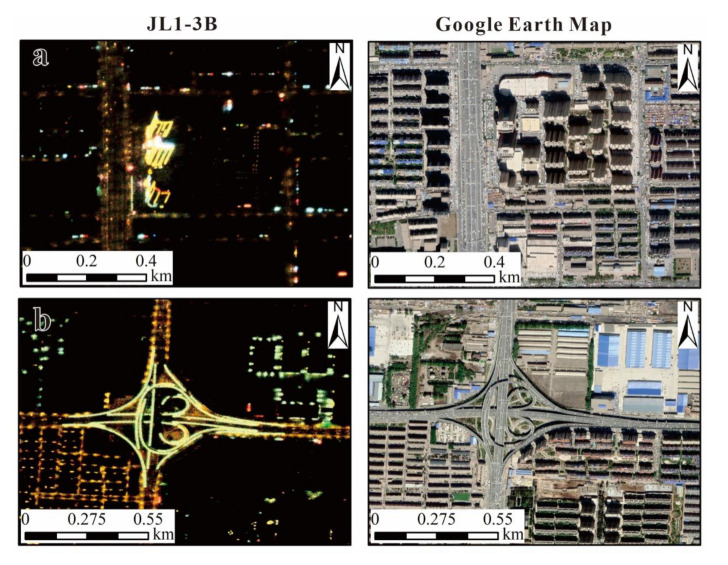
Limitations of JL1-3B NTL data: JL1-3B images (left panel), Google earth map (right panel) of two selected regions, including major building area (**a**), traffic lanes (**b**).

As the first commercial satellite to offer high-resolution, multispectral NTL data, JL1-3B had performed well in the RF regression model, but it still has some weaknesses. The area coverage of a single JL1-3B image is small in comparison to typical large study areas; furthermore, the data are not free and must be ordered. Therefore, it is expensive to purchase a large number of JL1-3B images to conduct a large-area study or a multi-temporal study. In this study, we selected ten JL1-3B images to ensure that the mosaic of them could cover the central urban area of Changchun. In addition, there are some limitations of the light detection ability of JL1-3B data; the radiance limit is $7\times 10^{-7}$ W/cm^2^/sr. As a result, some dim light cannot be detected. Figure 11a shows one selected region of buildings; it can be seen that almost no light was detected on the tops of the buildings. Last but not the least, the parcel-level datasets were generated by regarding the OSM as natural borders in this study, which caused amounts of illumination information inside the roads to be lost, as shown in Figure 11b.

Based on the research described above, we believed that there are some aspects that can be improved in future research:

(1) In this paper, 17 kinds of land features are selected to establish regression models for the land radiance. However, the forms of human activities are diverse. So, more types of features as variables should be taken into consideration for regression, such as population, gross national product, electricity, etc.
(2) Researchers ought to make full use of the multispectral advantages of JL1-3B data to extract information and recognize ground targets.
(3) At present, the NTL data are still acquired from satellites, so the same area will not be repeatedly observed in the short term. In future research, a small study area can be measured repeatedly over a long time to increase the temporal frequency of acquiring images, so as to analyze the NTL time series. For example, night-light sensors mounted on drones can be used to photograph NTL images of research areas [61].

## 6. Conclusions

JL1-3B and Luojia 1-01 are the new generation of NTL satellites. JL1-3B has higher spatial resolution and multispectral capability compared with Luojia 1-01. It shows great potential in the research field related to urban issues. In this paper, the road networks data were regarded as the segmentation boundary to generate parcels according to the Gini feature importance, the land features that have an impact on the radiance of NTL data were screened, NDVI and 16 important land features were selected from the land use types, and POIs were taken as independent variables eventually. The radiance of NTL data was regarded as a dependent variable to establish the RF regression model at the parcel level, revealing quantitatively the relationship between the radiance of NTL data of both JL1-3B and Luojia 1-01 NTL data and land features, respectively. Furthermore, the contribution of different land features was calculated. In addition, we analyzed the causes of radiance changes from human activities on a small scale and evaluated the performance of two NTL data sources in the RF regression process. The conclusions are as follows:

- After feature screening, 17 kinds of features were selected for regression to obtain the RF regression model with the best prediction ability. The oob scores corresponding to JL1-3B and Luojia 1-01 data are 0.9054 and 0.8304 respectively, which indicates that the established regression models of radiance intensity are quite accurate.
- In the two regression models corresponding to Luojia 1-01 and JL1-3B data, artificial buildings are the most important features, and the feature importance is 69.42% and 70.04%, respectively.
  In the land use types, the top two most important features are artificial buildings and cultivated land, with the importance of features being 70.04% and 20.04% for JL1-3B and 69.42% and 11.87% for Luojia 1-01, respectively. NDVI is of the third-most importance, which was 3.09% and 8.12% for JL1-3B and Luojia 1-01 data, respectively. In the POI types, the top two most important features are road ancillary facilities and food, with the importance of features being 0.77% and 0.81% for JL1-3B and 3.16% and 1.52% for Luojia 1-01 data, respectively.
- The contribution of different features to two kinds of NTL data is calculated, and several important features were stress-analyzed. Since the resolution of JL1-3B data is much higher than that of Luojia 1-01, the changes of the features contributions of the two are similar in large parcels, while they are significantly different in small parcels. Moreover, compared with Luojia 1-01 data, JL1-3B data are less affected by light overflow effect and saturation.
- By means of the RF regression algorithm, using JL1-3B data and Luojia 1-01 data, the relationship between the radiance value and the related land features in the study area was obtained. By analyzing the importance and contribution value of different features, this paper explores the influence of land features on night light. It also fully demonstrates the great potential of JL1-3B, a new generation of high spatial resolution and multispectral night data, in the study of human activities and urbanization processes, and it provides a reference for future related research.

## Figures and Tables

**Table 1:** Relevant parameters for different nighttime lights (NTL) images.

NTL Imagery	Spatial Resolution (m/pixel)	Launch	Spectral Band (nm)	Bit Depth
DMSP/OLS	3000	1992	400–1100 (panchromatic)	8 bits
VIIRS/DNB	740	October, 2011	505–890 (panchromatic)	14 bits
Luojia 1-01	130	June, 2018	460–980 (panchromatic)	14 bits
JL1-3B	0.9	January, 2017	430–512 (blue)	8 bits
			489–585 (green)	
			580–720 (red)	

**Table 2:** Details of data used in this study.

Data Name	Data Description	Time	Source
JL1-3B	NTL data with a spatial resolution of about 0.92 m	April, 2018	Provided by ChangGuang Satellite Technology Co., Ltd.
Luojia 1-01	NTL data with a spatial resolution of about 130 m	August, 2018	Hubei Data and Application Network of High Resolution Earth Observation System, Available online: http://www.hbeos.org.cn/
Road networks	Different levels of road vector data networks	2020	Open street map, Available online: https://www.openstreetmap.org/
POI	Point of Information	2020	Baidu Map Open Platform
Land Use Maps	Land use maps with a 30 m resolution	2010	GLOBELAND30. Available online: http://globallandcover.com/GLC30Download/index.aspx
Landsat8 OLI	Multispectral image	2017	United States Geological Survey, Available online: https://earthexplorer.usgs.gov/
Administrative Boundaries	Vector file of provinces and prefectures in study area	2017	National Geomatics Center of China: Available online: http://ngcc.sbsm.gov.cn/ngcc/

**Table 3:** The JL1-3B parameters for converting DN to radiance (W/m^-2^/sr^−1^).

Bands	a	b
R	9681	−4.73
G	5455	−3.703
B	2997	−4.471

**Table 4:** The Gini of different features importance (^1^: from land use, ^2^: from POI, ^3^: from NDVI).

ID	Feature	Luojia 1-01	JL1 -3B
1	Artificial surfaces ^1^	0.6942	0.7004
2	Cultivated land ^1^	0.1187	0.2044
3	NDVI ^3^	0.0812	0.0309
4	Road ancillary facilities ^2^	0.0316	0.0077
5	Grass lands ^1^	0.0194	0.0221
6	Food ^2^	0.0152	0.0081
7	Enterprises ^2^	0.0098	0.0072
8	Government agencies ^2^	0.0086	0.0029
9	Forests ^1^	0.0072	0.0078
10	Life services ^2^	0.0054	0.0029
11	Automobile maintenance ^2^	0.0019	0.0011
12	Automobile services ^2^	0.0019	0.0010
13	Automobile sales ^2^	0.0012	0.0018
14	Water bodies ^1^	0.0011	0.0005
15	Science and education ^2^	0.0010	0.0003
16	Financial insurance services ^2^	0.0008	0.0006
17	Shopping services ^2^	0.0007	0.0003

**Table 5:** Optimal RF regression model parameters values.

ID	Parameter	Optimal Value (JL1-3B)	Optimal Value (Luojia 1-01)
1	N_estimators	250	270
2	Max_features	10	11
3	Min_samples_leaf	14	7
4	Max_depth	9	8
5	Min_samples_split	14	7
